# Contractile effects of retatrutide in isolated mouse atrial preparations

**DOI:** 10.1007/s00210-025-04335-0

**Published:** 2025-06-04

**Authors:** Joachim Neumann, Undine Ahlrep, Britt Hofmann, Ulrich Gergs

**Affiliations:** 1 Institute for Pharmacology and Toxicology, Medical Faculty, Martin-Luther-University Halle-Wittenberg, Magdeburger Straße 4, 06097 Halle (Saale), Germany; 2 Department of Cardiac Surgery, Mid-German Heart Centre, University Hospital Halle, Ernst-Grube Straße 40, 06097 Halle (Saale), Germany

**Keywords:** GLP-1, Glucagon receptor, GIP, Retatrutide, Mouse atrium

## Abstract

When retatrutide stimulates the glucagon receptor (GCGR), the glucose-dependent insulinotropic polypeptide (GIP) receptor (GIPR), and the glucagon-like peptide-1 receptor (GLP-1R), then 3′,5′cyclic adenosine monophosphate (cAMP) is increased. We tested the hypothesis that retatrutide like the β-adrenoceptor agonist isoprenaline raises force of contraction (FOC) in isolated electrically driven (1 Hz) left atrial preparations (LA) and exerts positive chronotropic effects (PCE) in isolated spontaneously beating right atrial preparations (RA) from adult CD1 mice. While 100 nM isoprenaline increased FOC, retatrutide (100 nM) failed to increase FOC in LA. In isolated mouse right atrial preparations (RA), retatrutide exerted PCE that were potentiated by 100 nM rolipram but that were antagonized by adomeglivant, a GCGR antagonist. The PCE of retatrutide but not the PCE of isoprenaline were attenuated by H89, an inhibitor of the cAMP-dependent protein kinase (PKA). The PCE of retatrutide were not weakened by the β-adrenoceptor antagonist propranolol (1 µM) but were blocked by 1 µM carbachol, an agonist at M_2_-cholinoceptor, and this effect was reversed by 1 µM atropine, a muscarinic receptor antagonist. Likewise, the PCE of retatrutide were blocked by 1 µM (-)-N^6^-phenylisopropyladenosine (PIA), an A_1_-adenosine receptor agonist, and this effect was reversed by 1 µM DPCPX, an adenosine A_1_-receptor antagonist. We conclude that retatrutide excites the beating rate in RA via GCGR, signalling via cAMP and PKA. Isoprenaline and retatrutide might increase cAMP in different compartments of the mouse sinus node.

## Introduction

Retatrutide is under development as a drug to treat diabetes type 2 and obesity (Jastreboff et al. 2022). Retatrutide could activate GCGR, GIPR, and GLP1-R in cells transfected with these receptors (Coskun et al. 2022). More specifically, retatrutide increased cAMP in these receptor-transfected cells with affinities of (in nanomolar concentrations) 5.8, 0.06, and 0.78 at GCGR, GIPR, and GLP1-R, respectively (Coskun et al. 2022, Fig. 1, Table 1). It is accepted that agonists at GLP1-R reduce body weight, in part by reducing craving for food in the brain (Ryan 2022). GLP-1R also can be useful in diabetes type 2 because they reduce the release of glucagon from pancreatic cells (Ryan 2022). More recently, it became clear that also agonists at GCGR (Jastreboff et al. 2023; Neumann et al. 2023a, Neumann et al. 2023b) or at GIPR might reduce blood glucose levels and might therefore be useful in diabetes type 2 (Ussher et al. 2018). From studies in mice, it was made clear that beneficial effects of stimulation of GCGR, GIPR, and GLP1-R are indeed specific because they were deleted in the appropriate receptor knock out mice (Ali et al. 2014, Baggio et al. 2017, Ussher et al. 2018). Further work demonstrated that the effect on diabetes and on weight loss were additive, if one stimulated not one but two of these receptors. The next level is now reached with retatrutide that stimulates all three above mentioned receptors (Coskun et al. 2022).

**Fig. 1:**
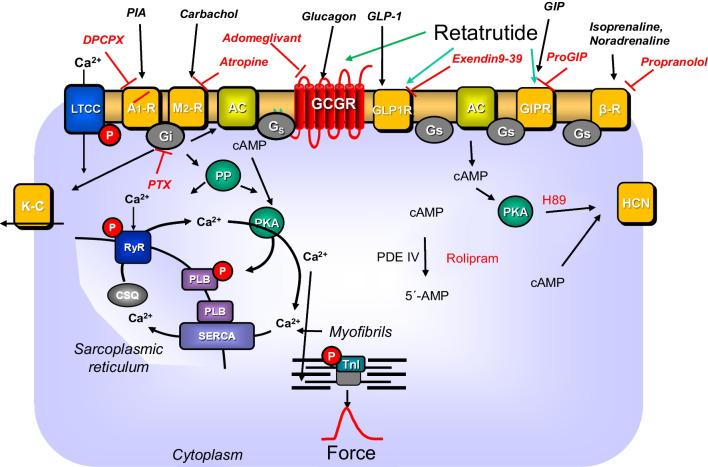
Conceivable actions of retatrutide in cardiomyocytes. Glucagon stimulates glucagon receptors (GCGR antagonized by adomeglivant). GLP-1 stimulates GLP-1 receptors (GLP-1R, antagonized by exendin9-39) and GIP the GIP receptors (GIPR, antagonized by ProGIP). Then via stimulatory GTP-binding proteins, adenylyl cyclase (AC) catalyzes the formation of cAMP which could directly activate hyperpolarization-activated cyclic nucleotide gated channels (HCN). The cAMP activates a cAMP-dependent protein kinase (PKA). This PKA activates by phosphorylation (P) cardiac regulatory proteins which are reversed by protein phosphatases (PP). The cAMP is degraded by phosphodiesterases IV (PDE) that can be inhibited by rolipram. PKA (inhibited by H89) can phosphorylate phospholamban (PLB) or HCN. SERCA pumps Ca^2+^ into the sarcoplasmic reticulum. Ca^2+^ binds to calsequestrin (CSQ). RyR indicates the ryanodine receptor. LTCC means the L-type Ca^2+^ channel. PIA (R-PIA) is an agonist at A_1_-adenosine receptors (A_1_-R) which is antagonized by DPCPX. Carbachol stimulates the M_2_-muscarinic receptor (M_2_-R) which is antagonized by atropine. The effect of carbachol and PIA are mediated by inhibitory GTP-binding proteins (G_i_) which are inactivated by pertussis toxin (PTX). The subunits of G_i_ may inhibit AC and/or open potassium channels (K-C) and/or PP. Isoprenaline or noradrenaline stimulate the β-adrenoceptors (β-R) which is antagonized by propranolol. Myofibrils are responsible for the generation of force which is symbolized here by a single muscle contraction over time

**Table 1:** Overview of the drugs used in the present study. We list the targets of the drugs and their putative mechanism of action

Drug	Target	Mechanism
Glucagon	Glucagon receptor (GCGR)	Agonist
Glucagon-like-protein-1(GLP-1)	Glucagon-like-protein-1 receptor (GLP-1R)	Agonist
Glucose-dependent insulinotropic polypeptide (GIP)	Glucose-dependent insulinotropic polypeptide (GIPR)	Agonist
N-[2-(p-Bromocinnamylamino)ethyl]−5-isoquinolinesulfonamide (H89)	cAMP-dependent protein kinase (PKA)	Inhibitor
Adomeglivant	Glucagon receptor (GCGR)	Antagonist
Pro(3)GIP (ProGIP)	Glucose-dependent insulinotropic polypeptide (GIPR)	Antagonist
Exendin9-39 (exendin)	Glucagon like protein 1 receptor (GLP-1R)	Antagonist
Carbachol	M-cholinoceptor (M_2_-R)	Agonist
Atropine	M-cholinoceptor (M_2_-R)	Antagonist
R-N^6^−2-phenylisopropyladenosine (PIA)	A_1_-adenosine receptor (A_1_-R)	Agonist
8-Cyclopentyl-1,3-dipropylxanthine (DPCPX)	A_1_-adenosine receptor (A_1_-R)	Antagonist
Isoprenaline	β-Adrenoceptor (β-R)	Agonist
Propranolol	β-Adrenoceptor (β-R)	Antagonist
Rolipram	Phosphodiesterase III (PDE III)	Inhibitor
Noradrenaline	Adrenoceptor	Agonist

The target of drugs to treat diabetes or reduce body weight are not cardiac cells. Indeed, side effects on the heart can be deleterious in drugs that reduce body weight. Two examples might suffice here: fenfluramine was used in the past as an anorectic agent acting, in part, in the brain. However, fenfluramine was removed from the market when it became clear that fenfluramine by stimulating 5-HT2 serotonin receptor in the heart could lead to fibrosis and thus to deadly valvular heart disease (Neumann et al. 2023d; Surapaneni et al. 2011). Amphetamines are still used to reduce body weight by acting mainly in the central nervous system. However, amphetamines are indirect sympathomimetic drugs and lead to death, e.g., by inducing cardiac arrhythmias (Neumann et al. 2023d, Ryan 2022). Hence, there is precedence that weight loss drugs can have detrimental cardiac effects and this thought initiated the present study.

In the mouse heart, all three receptors, GCGR, GIPR, and GLP-1R, have been detected, at least at the mRNA level (Ali et al. 2014, Baggio et al. 2017, Ussher et al. 2018). Hence, it was conceivable that retatrutide could exert effects on force of contraction (FOC) or positive chronotropic effects (PCE) via any of these receptors.

In humans, retatrutide in safety studies elevated the heart rate and decreased the blood pressure (Coskun et al. 2022, Jastreboff et al. 2023). Firstly, this may result from direct PCE of retatrutide on the human cardiac sinus node via GCGR and/or GIPR and/or GLP1-R. Secondly, retatrutide may have released cardiac noradrenaline in the heart to raise the beating rate through cardiac β-adrenoceptors. Thirdly, retatrutide may have dilated arterial vessels via GCGR and/or GIPR and/or GLP1-R, and subsequently, a compensatory reflex tachycardia might have ensued.

Hence, we started the present study to find out whether or not retatrutide can affect directly the mechanical function of the mammalian heart, using mouse atrial preparations as our model system. Mice are often used when the effects of a stimulation of GCGR, GLP-1R, or GIPR are studied (e.g., Ali et al. 2014, Baggio et al. 2017, Ussher et al. 2018). More specifically, mice have also been used for the study of metabolic effects of retatrutide (Coskun et al. 2022, Cui et al. 2025, Ma et al. 2025). However, we already demonstrated that in the mouse atrial preparations, GLP1-receptor agonists (exenatide, liraglutide, semaglutide) did not increase FOC or beating rate (Neumann et al. 2024a). Likewise, GIP failed to increase FOC or the beating rate in isolated mouse atrial preparations (Neumann et al. 2024b). Glucagon itself failed to increase FOC but exerted a potent positive chronotropic effect in mouse atrial preparations (Neumann et al. 2023a, c, d, e; Neumann et al. 2024a, 2024b, 2025). Hence, it was an open question how a triple receptor agonist like retatrutide would act in on beating rate in RA and on force of contraction in LA. Preliminary results have been reported in an abstract form (Ahlrep et al. 2025).

Thence, we tested the following hypotheses:

1. Retatrutide increases the beating rate mouse right atrial preparations.
2. Retatrutide augments force of contraction in mouse left atrial preparations.
3. These contractile effects of retatrutide are mediated by GIPR.
4. These contractile effects of retatrutide are mediated by GLP-1R.
5. These contractile effects of retatrutide are mediated by GCGR.

## Material and methods

### Contractile studies in mice

Housing (in the animal facility of our medical faculty), handling, raising, and sacrifice of mice complied with local regulations (permission TS 10–24). Mice were subjected to cervical dislocation before the thorax was opened. The heart as a whole was prepared and moved to a glass Petri dish filled with a modified Tyrode’s solution, kept at room temperature. In this buffer, the right (RA) or left atrial preparations (LA) from the mice (wild type: CD1, random sex, about 120 days of age) were isolated. Then, LA and RA were mounted in organ baths (containing 10 ml buffer volume) as previously described (e.g., Gergs et al. 2017). The modified Tyrode’s solution contained in milli molar concentrations (mM): 119.8 NaCI, 5.4 KCI, 1.8 CaCl_2_, 1.05 MgCl_2_, 0.42 NaH_2_PO_4_, 22.6 NaHCO_3_, 0.05 Na_2_EDTA, 0.28 ascorbic acid, and 5.05 glucose. Ascorbic acid is used here as an antioxidant to maintain the activity of, for instance, isoprenaline. The solution was continuously gassed with 95% O_2_ and 5% CO_2_ and maintained at 37 °C and pH 7.4 in the organ baths. Spontaneously beating RA were used to study any chronotropic effects. LA were used to study force under isometric conditions. Atria were stretched to optimal length that allowed maximal generation of FOC. LA were stimulated (60 beats per minute, bpm) electrically with platinum electrodes with rectangular impulses of direct currents from a Grass stimulator SD 9 (Quincy, Massachusetts, USA). Voltage ranged between 5 and 10 Volts, just sufficient to initiate contractions. Electrical impulses had a length of 5 ms. The signals from the force transducer (Hugo Sachs, Freiburg, Germany) were fed into a bridge amplifier, digitized, and stored on a commercial personal computer. The signals were quantified using a commercial software (Lab Chart 8 from ADInstruments bought through their distributor in Oxford, England). In some experiments, we finally applied receptors antagonists to the organ baths or the β-adrenoceptor agonist isoprenaline as a positive control, as delineated in the figure legends.

### Data analysis

Data shown are mean ± standard error of the mean. Statistical significance was estimated using Student’s *t*-test or the analysis of variance followed by Bonferroni’s *t*-test as described in the legends. A *p*-value < 0.05 was considered to be significant.

### Drugs and materials

Retatrutide was from Biomol (Hamburg, Germany) and was dissolved in water at 10 mM. Pro(3)GIP, exendin 9–39, all dissolved in water at 1 mM, came from Bachem (Bubendorf, Switzerland). DPCPX (8-cyclopentyl-1,3-dipropylxanthine, dissolved in dimethylsulfoxide, 10 mM) was from Tocris (Bristol, England). Carbachol hydrochloride (10 mM dissolved in water) (-), isoprenaline tartrate (100 mM, dissolved in water), R-PIA (R-N^6^−2-phenylisopropyladenosine (10 mM, dissolved in dimethylsulfoxide), N-[2-(p-bromocinnamylamino)ethyl]−5-isoquinolinesulfonamide dihydrochloride (H89,10 mM, dissolved in water), and atropine hydrochloride (10 mM, dissolved in water) were from Merck (Dreieich, Germany). All other chemicals were of the highest purity grade commercially available. Deionized water was used throughout the experiments to prepare a modified Tyrode’s solution. Stock solutions were prepared fresh daily.

We used as final bath concentration 100 nM exendin9-39 and 100 nM ProGIP based on our contraction data in HAP (Neumann et al. 2023a, 2024a, 2024b). These concentrations were higher than the affinity constants at the receptors of interest and therefore should have been adequate (Neumann et al. 2023b). The concentrations (1 µM each) of carbachol, R-PIA, atropine, and DPCPX were based on our own work (Neumann et al. 1999, 2003; Schwarz et al. 2023, 2024, 2025).

## Results

**Fig. 2:**
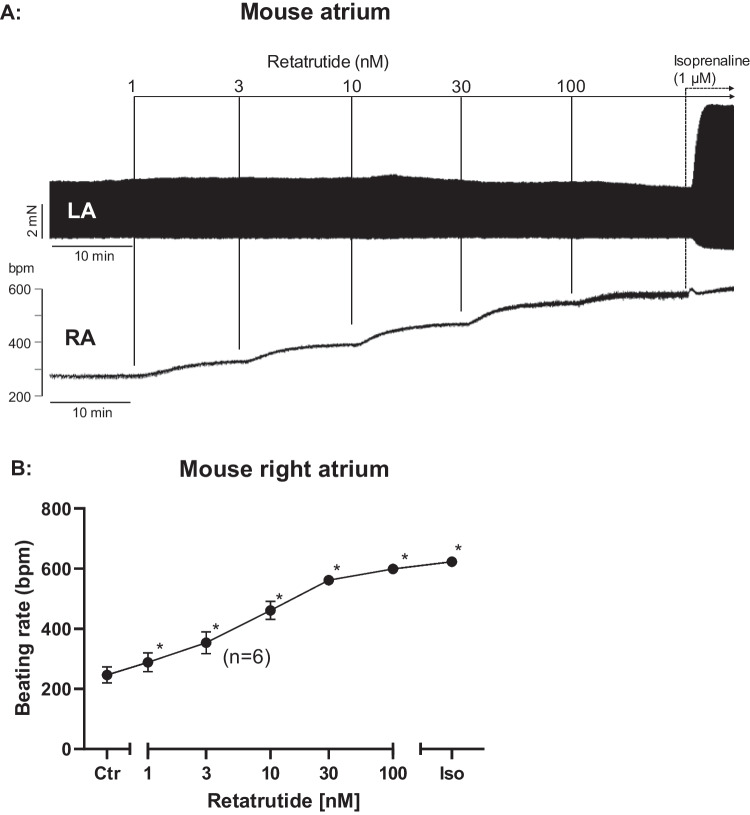
Retatrutide increases the beating rate. **A** Original recording of FOC in mouse left electrically stimulated (top, LA) and right spontaneously beating (bottom, RA) isolated atrial preparations. Additions of retatrutide (cumulatively applied) and isoprenaline are indicated with vertical lines. Vertical bar as beginning of (**A**) (top) gives force of contraction in milli Newton (mN). Ordinate (bottom) gives beating rate in beats per minute (bpm). Horizontal bars give time in minutes (min). **B** Summarized effects of retatrutide and 1 µM isoprenaline on beating rate in RA. Ordinate gives beats per minute (bpm). Abscissa: negative decadic logarithm of concentration of retatrutide or effect of isoprenaline (Iso) or pre-drug value (Ctr). Number in brackets indicate number of experiments

We first studied the effect of retatrutide cumulatively applied on force of contraction in LA preparations. However, while 100 nM retatrutide increased FOC in isolated electrically stimulated human atrial preparations (Ahlrep et al. 2025) and increased beating rate in RA (Fig. 2A bottom), we failed to detect any PIE in LA (Fig. 2A, top). This finding is depicted as an original recording in Fig. 2A (top). As a positive control, we applied isoprenaline (Fig. 2A, top). One discerns in Fig. 2A (top) that 1 µM isoprenaline was very effective to raise FOC in a mouse atrial preparation where retatrutide was ineffective (Fig. 2A). This lack of a PIE to retatrutide was unexpected as retatrutide and isoprenaline in principle were thought to use the same second messenger, namely, cAMP (Fig. 1, Table 1).

However, retatrutide was not completely without effects in the mouse atrium. Focusing on the RA, one can see that retatrutide time- and concentration-dependently increased the beating rate (Fig. 2A, bottom). Then, we repeated these experiments to obtain summarized data (Fig. 2B). This positive chronotropic effect gained significance at 3 nM retatrutide (Fig. 2B). A 100 nM retatrutide was nearly as effective as 1 µM isoprenaline to raise beating rate (Fig. 2A, bottom, Fig. 2B).

**Fig. 3:**
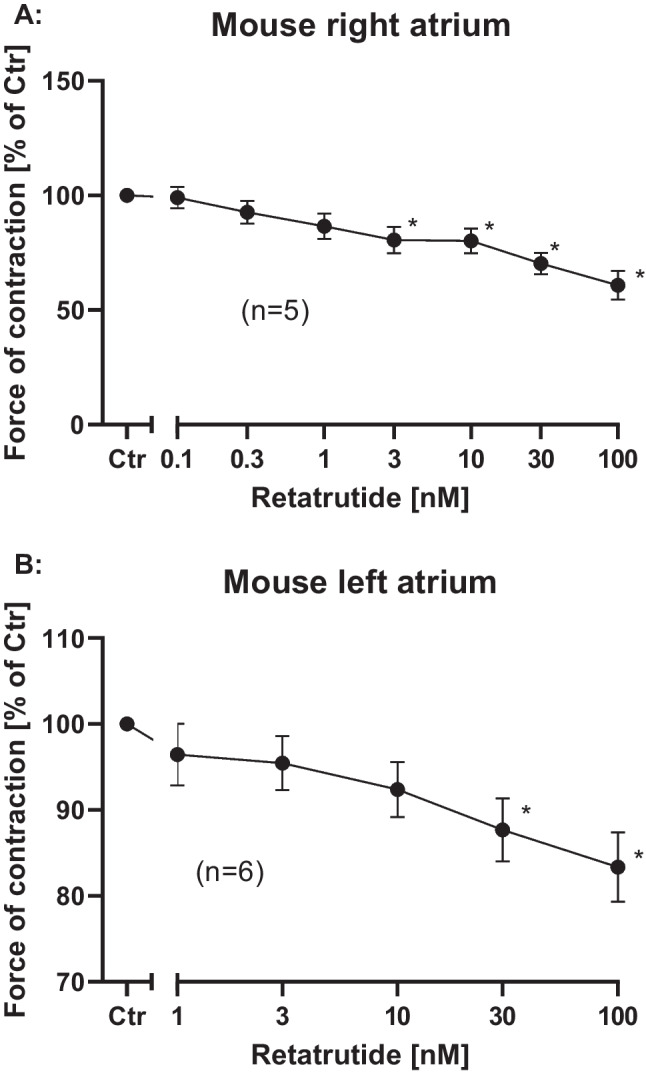
Retatrutide fails to increase force of contraction. Force of contraction was measured in isolated spontaneously beating right atrial mouse preparations (**A**) or in electrically stimulated mouse left atrial preparations (**B**). Ordinates indicates force of contraction in percentage of pre-drug value (Ctr). Abscissa gives concentration of retatrutide in nM concentrations. Number in brackets indicates number of experiments. * indicate *p* < 0.05 against Ctr

As concerns the FOC in RA, we noted a concentration-dependent decline in FOC in RA starting at 30 nM retatrutide (Fig. 3A). These effects are in all likelihood indirect: in the mouse atrium, an increase in beating rate per se reduces FOC, probably of depressed release of Ca^2+^ from the sarcoplasmic reticulum (Stemmer and Akera 1986). Please not further that we used different scales for the ordinates in Fig. 3A, B to make differences easier to detect. As concerns FOC in LA, we noted with a cumulative addition of retatrutide a negative inotropic effect, but only at high concentrations of retatrutide (Fig. 3B). This clearly shows that retatrutide, although it can increase cAMP in other cell types, does not increase as we hypothesized for the FOC in LA. The small decrease in FOC in LA at 30 nM and 100 nM is in our view a run down of the LA over the time of the experiments (compare our previous data in wild type LA: e.g., Gergs et al. 2019).

**Fig. 4:**
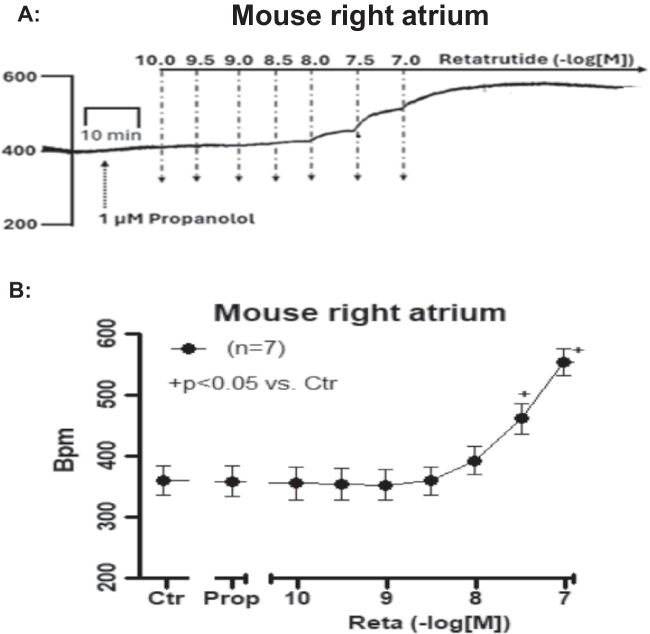
Propranolol does not block the effect of retatrutide. **A** An original recording of the beating rate in the spontaneously beating right atrium. Horizontal bar indicates time in 10 min. Arrows indicates where 1 µM propranolol was added. Concentration of retatrutide in decadic logarithm of molar concentration in is indicated by a horizonal line above the original recording. **B** Summarized concentration dependent effects of retatrutide on beating rate. Abscissa indicated the concentration of retatrutide in negative decadic logarithm of molar concentrations or control values (before any drug application: Ctr). Number in brackets indicates number of experiments. + indicate *p* < 0.05 against Ctr. Ordinates indicates beating rate in beats per minute

The question arose via which receptor retatrutide might lead to a positive chronotropic effect. Hence, we first wanted to study a possible involvement of the β-adrenoceptor (Fig. 1). This was done in part, because ancient data found that glucagon (the physiological agonist at GCGR) could release noradrenaline in the heart and this noradrenaline would the stimulate β-adrenoceptors (Fig. 1, Farah and Tuttle 1960). Hence, we gave first 1 µM propranolol (to block any action of endogenous noradrenaline at stimulate β-adrenoceptors (Fig. 1), then we added cumulatively retatrutide (original recording: Fig. 4A). It is apparent that the positive chronotropic effects of retatrutide are not abolished by propranolol (Fig. 4A). These data are summarized in Fig. 4B.

**Fig. 5:**
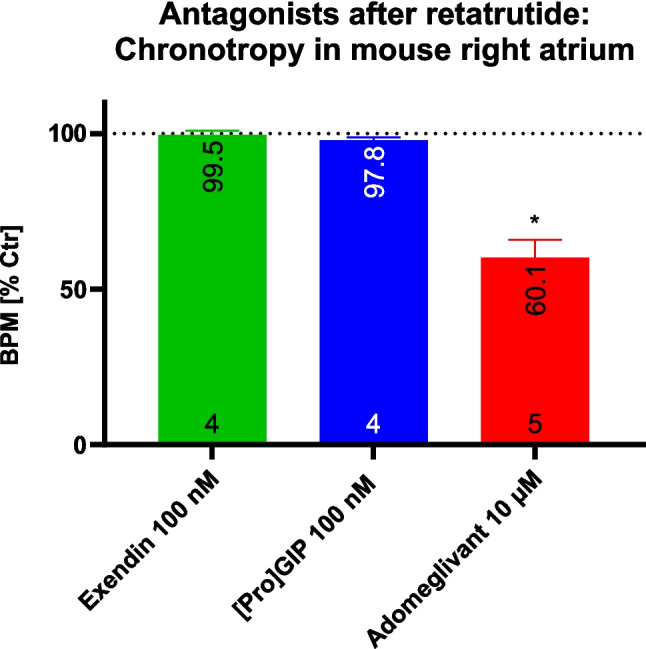
Effect of retatrutide on beating rate is mediated by GCGR. The beating rate before the exendin 9–39 (exendin) or ProGIP or adomeglivant was defined as 100% and regarded as control (Ctr) as seen in Fig. 4. Thereafter the antagonist was added, and its maximim negative inotropic effect was measured. Several such experiments are summarized. Numbers in bars: top numbers: arithmetic mean values of beating rate; bottom numbers: number of experiments. *p < 0.05 vs. Ctr

Now that we had ruled out an involvement of the β-adrenoceptor in the PCE of retatrutide, the question intensified which receptor mediates the PCE of retatrutide, three receptors remained (Table 1, Fig. 1), namely, the GCGR, the GIPR, and the GIP-1R. Hence, after we established full cumulative concentration response curves for retatrutide, we gave exendin9-39, ProGIP, or adomeglivant. Only adomeglivant significantly reduced beating rate that had been stimulated by retatrutide (Fig. 5). Thus, we infer that the positive chronotropic effect of retatrutide in the mouse heart is GCGR-mediated (Fig. 5).

To better characterize how retatrutide via the GCGR leads PCE, we studied drugs that have a bradycardic effect, namely, PIA and carbachol (Fig. 1). PIA and carbachol may cause a NCE by inhibiting the activity of adenylyl cyclases that were augmented by GCGR-stimulation by retatrutide. Moreover, it might be of clinical relevance whether endogenous acetylcholine or adenosine via cardiac M_2_-cholinoceptors or via cardiac A_1_-adenosine receptors may reduce positive chronotropic effects of retatrutide in patients. Please consider that PIA and carbachol antagonize the PCE of isoprenaline which we compare here always with retatrutide (Neumann et al. 2003).

**Fig. 6:**
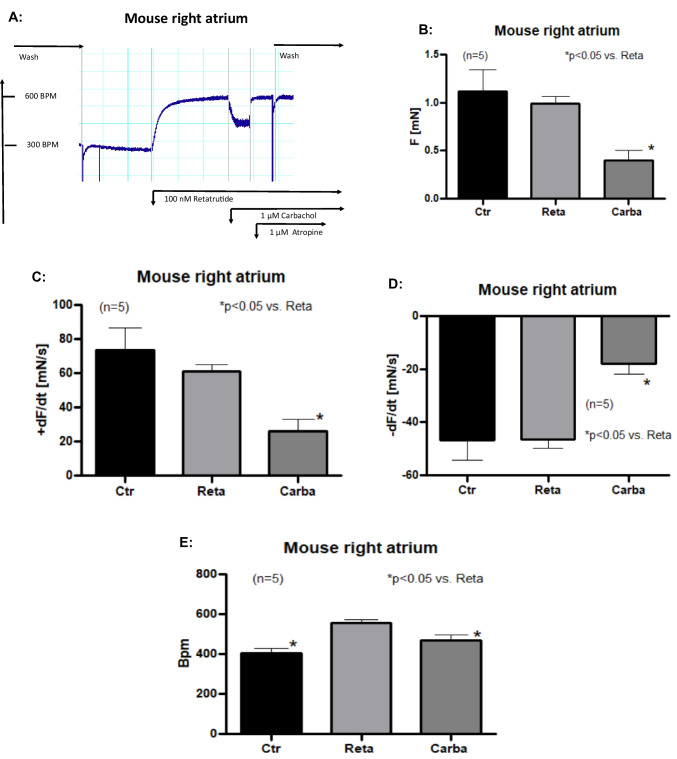
Carbachol reduces beating rate after retatrutide. **A** Original recording of beating rate in isolated spontaneously beating right atrial mouse preparations. Ordinate: beating rate in beats per minute. Horizontal bar indicates time in minutes (min). Arrows indicate addition of 100 nM retatrutide, 1 µM carbachol or 1 µM atropine, respectively. **B** Bar diagram of force of contraction in right atrial preparations. Ordinate (F) in mN. * indicates *p* < 0.05 versus retatrutide. Ctr: pre-drug value. Reta (100 nM retatrutide). Carba (1 µM carbachol). **C** Bar diagram of the maximum rate of tension development. Ordinate in mN/s. * indicates *p* < 0.05 versus retatrutide. Ctr: pre-drug value. pre-drug value. Reta (100 nM retatrutide). Carba (1 µM carbachol). **D** Bar diagram of rate of relaxation. Ordinate in mN/s. * indicates *p* < 0.05 versus retatrutide. Ctr: pre-drug value. pre-drug value. Reta (100 nM retatrutide). Carba (1 µM carbachol). **E** Bar diagram of the beating rate. Ordinate in beats per minute (Bpm). pre-drug value. Reta (100 nM retatrutide). Carba (1 µM carbachol). * indicates *p* < 0.05 versus retatrutide (Reta). Number in brackets indicates number of experiments

Thus, we first stimulated isolated right atrial preparations with 100 nM retatrutide and then when the PCE had gained plateau, we added carbachol. We see in an original tracing (Fig. 6A) that carbachol reduced the retatrutide-stimulated beating rate, rapidly and effectively. We used 1 µM carbachol to reduced beating rate in RA based on many previous reports from our group (e.g., Neumann et al. 2003). This negative chronotropic effect of carbachol is not simply due to a run down of the sample but is receptor mediated because addition of atropine rapidly reversed the negative chronotropic effect of carbachol to about pre-drug values (Fig. 6A). This negative chronotropic effect of carbachol from several similar experiments is significant as exemplified in Fig. 6E). This negative chronotropic effect of carbachol was accompanied by a negative inotropic effect (Fig. 6B). Similarly, the rate of tension development (Fig. 6C) and the rate of relaxation were significantly reduced by carbachol after retatrutide (Fig. 6D).

**Fig. 7:**
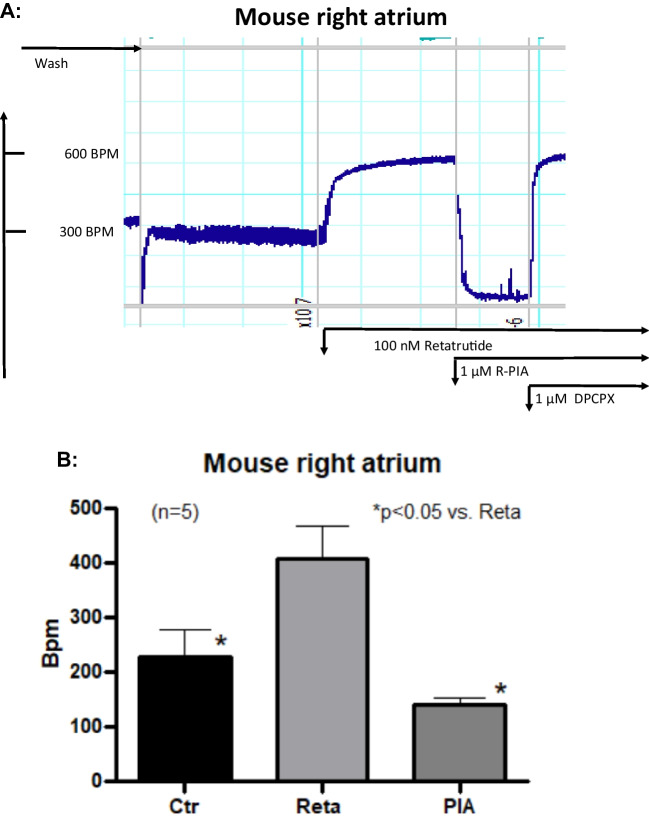
PIA reduces beating rate after retatrutide. **A** Original recording of beating rate in isolated spontaneously beating right atrial mouse preparations. Ordinate: beating rate in beats per minute (BPM). Horizontal bar indicates time in minutes (min). Arrows indicate addition of 100 nM retatrutide, 1 µM PIA and DPCPX, respectively. **B** Bar diagram of the beating rate. Ordinate gives beats per minute (Bpm). Ctr: pre-drug value. Reta (100 nM retatrutide). PIA (1 µM PIA). * indicates *p* < 0.05 versus (Reta)

In a similar pattern, PIA reduced beating rate after stimulation with retatrutide. This is seen in an original recording (Fig. 7A): PIA rapidly and effectively reduced the beating rate that had been raised by 100 nM retatrutide. One sees that retatrutide increased beating rate and then additionally applied PIA reduced beating rate substantially and significantly (Fig. 7A). This negative chronotropic effect of PIA was rapidly reversed by addition of DPCPX. This is consistent with our assumption that R-PIA acted by stimulation of A1-adenosine receptors to reduce the positive chronotropic effects of retatrutide. Data for the negative chronotropic effect of PIA after retatrutide are summarized in Fig. 7B.

**Fig. 8:**
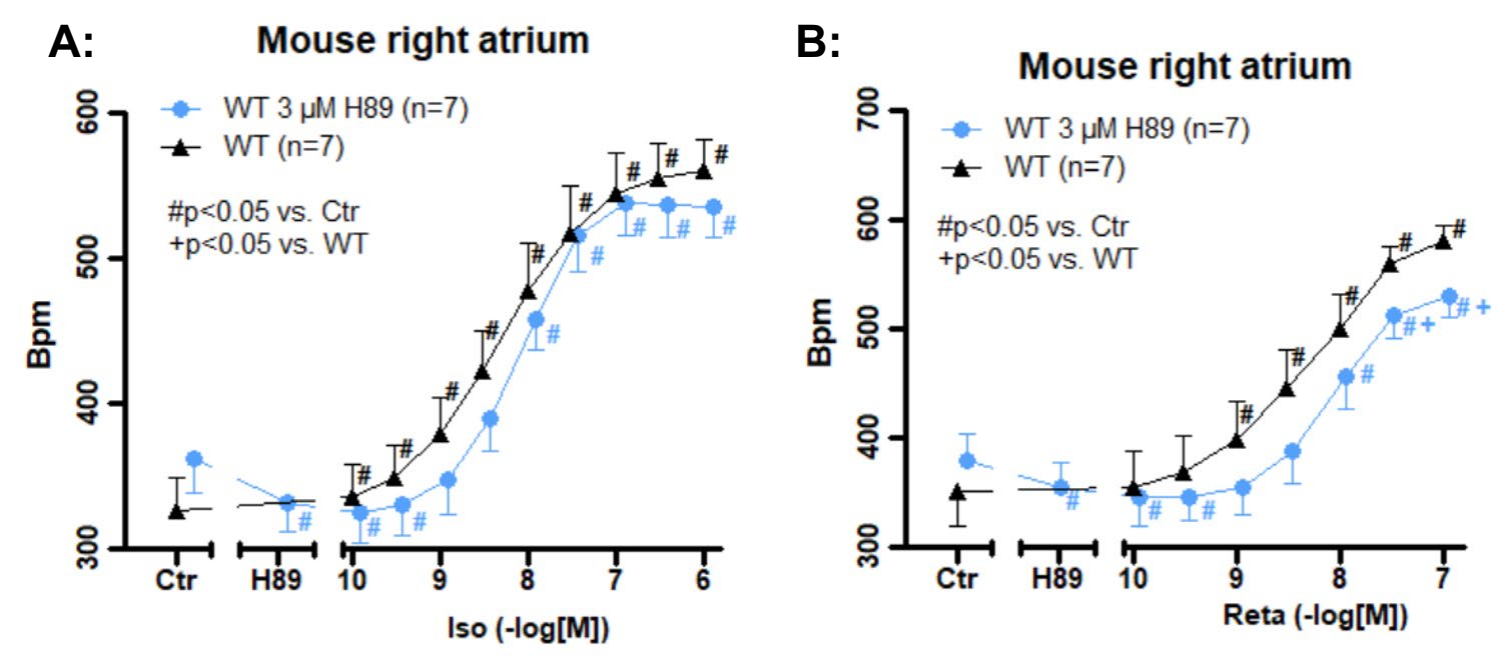
H89 attenuates the effect of retatrutide on beating rate. **A** Concentration response curve for the effect of isoprenaline (Iso) on beating rate in isolated mouse right atrial preparations (WT) in the presence (circles) or in the absence of 3 µM H89 (triangles). **B** Concentration response curve for the effect of isoprenaline on beating rate) in isolated right atrial preparations. Ordinates indicate beating rate in beats per minutes. * indicates the first significant (*p* < 0.05) difference versus Ctr (pre-drug value). Numbers in brackets indicate the number of experiments. Abscissae control conditions (Ctr: pre-drug) or after 3 µM H89 or indicate negative decadic logarithm of isoprenaline or retatrutide in molar concentration, which were subsequently applied. Number in brackets indicates number of experiments. # indicate *p* < 0.05 versus Ctr. + indicate *p* < 0.05 against in the absence of H89

Next, the question arose, if cAMP is involved in the PCE of retatrutide, and more specifically cAMP acting directly or via cAMP-dependent protein kinase (Fig. 1). To this end, we applied in further experiments the PKA inhibitor H89 which we used in the past in mouse atrial preparations (Gergs et al. 2019, Table 1). First, we studied isoprenaline as a comparator. It is generally thought that isoprenaline increases beating rate in mice independent of protein phosphorylation (discussed in Merino et al. 2015). Isoprenaline alone, as expected, exerted a concentration dependent PCE in RA (Fig. 8A). However, this PCE was not shifted to higher concentration of isoprenaline by H89, suggesting that PKA dependent phosphorylation of HCN under our experimental conditions does not take place (Fig. 1). However, under the same experimental conditions, it turned out that H89 shifted the PCE to larger concentrations of retatrutide (Fig. 8B). This suggests to us that cAMP formed after retatrutide stimulation in RA, acts via PKA in the sinus node of the mouse. Thence, signal transduction in the mouse sinus node is different between isoprenaline and retatrutide.

**Fig. 9:**
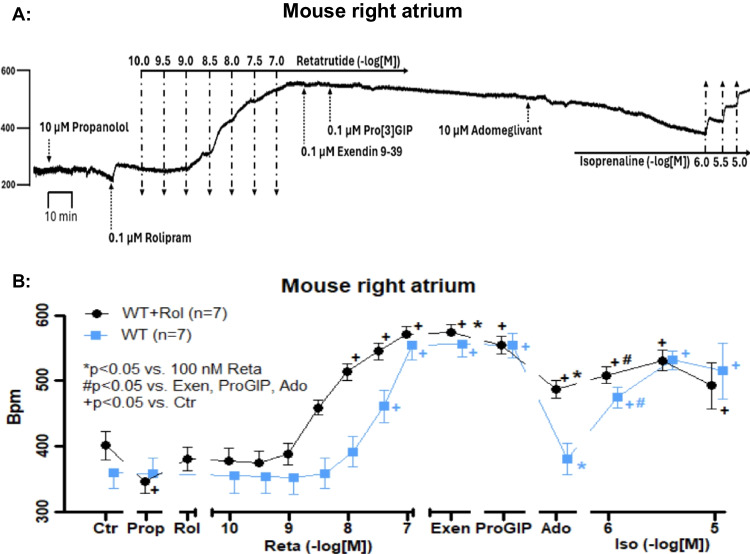
Rolipram potentiates the effect of retatrutide on beating rate. **A** Original recording of beating rate in isolated right atrial mouse preparations. Horizontal bar indicates time in 10 min. Arrows indicate addition of 10 µM propranolol then 100 nM rolipram, then retatrutide was cumulatively applied thereafter exendin9-39 (Exen), then ProGIP, and finally, 10 µM adomeglivant (Ado). At the end, increasing concentrations of isoprenaline were cumulatively applied. Please note that isoprenaline was here less effective than in previous figures, because the bath still contained 10 µM propranolol from the initial incubation. The concentration of retatrutide is given in negative decadic logarithm of molar concentrations as indicated by a horizontal line above the tracing. Horizontal line indicates time in minutes (min). **B** Summarized effect of retatrutide on beating rate in the absence (squares) and the presence of 100 nM rolipram (circles). Ctr indicates basal conditions, Prop means in the presence of 10 µM propranolol, Rol means in the presence of 100 nM rolipram. Abscissa otherwise indicates the negative logarithmic concentrations of retatrutide. Then, the effect of 100 nM exendin 9–39 (Exen), 100 nM ProGIP and 10 µM adomeglivant (Ado) are presented. Finally the effects of additional isoprenaline (Iso) are given in negative logarithmic concentrations. Ordinates give beats per minutes (BPM). Horizontal line indicates time in minutes (min). Numbers in bracket indicate the number of experiments. + indicates *p* < 0.5 vs. Ctr, * indicates *p* < 0.05 vs. 100 nM retatrutide (Reta), # indicates *p* < 0.05 vs. the maximum effects of Exen, ProGIP or Ado

To study the signal transduction of retatrutide via cAMP further, we thought that an inhibition of the phosphodiesterase III (inhibited by rolipram) that degrades cAMP in the sinus node of mice (Fig. 1) might potentiate the positive chronotropic effect of retatrutide. This turned out to be true. As seen in Fig. 9A, in the presence of rolipram, which exerted a small PCE of its own, the concentration response curve to retatrutide was shifted to the left (Fig. 9B). This suggest to us that the PCE of retatrutide is mediated by cAMP.

## Discussion

**Fig. 10:**
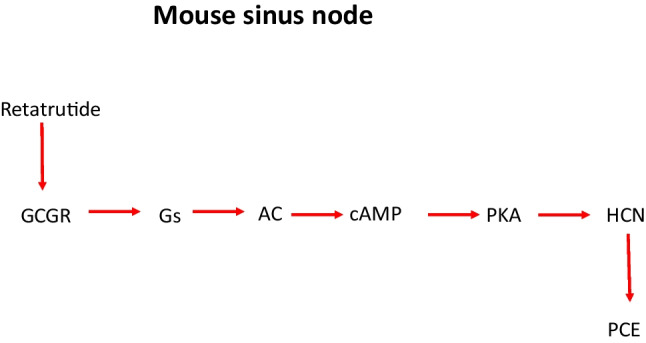
Signalling scheme for retatrutide. To summarize our interpretation of our findings, retatrutide binds to glucagon receptors (GCGR). Then, GCGR via stimulatory GTP-binding (Gs) the activity of adenylyl cyclase (AC). Hereby, cAMP is generated. This cAMP activates cAMP-dependent protein kinase (PKA). This activates HCN and this depolarizes the cardiomyocyte and as a consequence, then the beating rate in the mouse atrium increases (PCE)

The main new finding in this communication is that retatrutide exerts a PCE in RA. This PCE of retatrutide is GCGR-mediated (Fig. 10). This PCE of retatrutide involves a cAMP pool that activates PKA in the mouse sinus node cells (Fig. 10).

Why do we conclude that retatrutide increased the beating rate in mouse right atrial preparations probably via stimulation of GCGR? This is likely because retatrutide stimulates GCGR in cell culture work with transfected GCGR receptors and because the physiological agonist, namely, glucagon at GCGR can induce a positive chronotropic effect in mouse right atrial preparations (Neumann et al. 2023a; Schmidt et al. 2024). Moreover, the PCE of glucagon or retatrutide is attenuated by GCGR-antagonists (Neumann et al. 2023a; Schmidt et al. 2024, this report).

In contrast, we deem it unlikely that the PCE of retatrutide is mediated via GLP-1R or via GIPR. We write this, because the GLP-1 agonists semaglutide, liraglutide, and exenatide each failed to increase beating rate in RA (Neumann et al. 2023b, Neumann et al. 2024a). Hence, it is improbable that a GLP-1R agonist like retatrutide could raise the beating rate via GLP-1R. In addition the PCE of retatrutide was not blocked by exendin9-39 (Table 1). Likewise, GIP did not increase heart rate in mouse right atrial preparations (Neumann et al. 2024b). Moreover, the PCE of retatrutide was not reversed by ProGIP (Table 1). Thence, it is unlikely that retatrutide could raise the beating rate via GIPR. Thus, also by exclusion of other receptors, retatrutide most likely increased beating rate in RA via GCGR.

Moreover, our present data with retatrutide are in line with previous reports on contractile effects in the rat atrium. Glucagon in the isolated spontaneously beating rat right atrium increased the beating rate but not FOC (Merino et al. 2015). This effect was attenuated by protein kinase inhibitors like H89 which we also used in the present report (Merino et al. 2015). However, the PCE of isoprenaline in rat right atrium were not altered in their potency by the protein kinase inhibitor H89 (Merino et al. 2015). The authors argue that their data indicate that isoprenaline acts on a pool of cAMP that directly acts on its target protein (hyperpolarization-activated cyclic nucleotide gated channels, HCN) in the rat sinus node to raise the beating rate (Merino et al. 2015). In contrast, glucagon requires PKA and hypothetically a phosphorylation of HCN to exert a PCE (Merino et al. 2015). Their findings are also a framework to understand our present data with phosphodiesterase inhibitors: retatrutide increased the beating rate more in the presence of rolipram in mouse right atrial preparations. However, the effect of isoprenaline to raise the beating rate in right atrial preparation of mice was not potentiated by phosphodiesterase inhibitors in the present report. This also has already been reported by others in mice: the positive chronotropic effect of noradrenaline (which acts via β-adrenoceptor as isoprenaline, Fig. 1) was not potentiated by rolipram (Galindo-Tovar et al. 2016). Others suggested that glucagon and isoprenaline might activate a different compartment of cAMP at least in the sinus node of the rat (Merino et al. 2015). The same might be hypothesized to hold true in mice (Fig. 10): we might speculate that β-adrenoceptors and GCGR are located in the sarcolemma at different locations (compartments) in the mouse sinus node cells. Thence, we suggest that also in mouse the GCGR couples via a mechanism involving PKA to the beating rate in RA like in the rat. Thus, compartment would explain our findings with retatrutide, rolipram, and H89 on the beating rate in RA.

To further characterize the PCE of retatrutide, we used typical negative chronotropic interventions that are claimed to act through the adenylyl cyclase. Namely, we studied whether PIA, an A_1_-adenosine receptor-agonist, and carbachol, an M-cholinoceptor agonist, might attenuate the PCE of retatrutide in RA (Fig. 1, Table 1). We have noted before that after stimulation of the beating rate of the RA with isoprenaline (1 µM) at the concentration used here, 1 µM, PIA exerted a prominent negative chronotropic effect in isolated mouse right atrial preparations (e.g., Neumann et al. 1999, 2003). These anti-β-adrenergic effects of PIA or carbachol in mice are pertussis toxin-sensitive and are thence probably mediated by action of GTP-binding inhibitory proteins (Neumann et al. 2003, Fig. 1). Such pertussis sensitive proteins should mediate the negative chronotropic effects of carbachol or PIA firstly by inhibition of the activity of adenylyl cyclase (Fig. 1) and/or secondly via direct opening of cardiac potassium channels in the sinus node cells of the mouse right atrium (Fig. 1) or thirdly via activation of protein phosphatases (discussed in Schwarz et al. 2023, Fig. 1. Thus, the GCGR-mediated stimulation of cAMP content by retatrutide and the cAMP-induced stimulation of HCN might be reversed by inhibition of cAMP generation through M_2_-cholinceptors and/or A_1_-adenosine receptors. Another explanation is that carbachol and PIA open cardiac potassium channels in the sinus node cells to prolong the time for a new depolarization (Fig. 1). The reason for the lack of a positive inotropic effect (PIE) in RA can be readily understood if we assume that the negative staircase phenomenon is overcoming any PIE by an increase in cAMP in right atrial cardiomyocytes outside the sinus node (Stemmer and Akera 1986). The present data with retatrutide are supported by a previous report from our group, that the PCE of glucagon itself in RA are reversed by PIA and carbachol (Schmidt et al. 2024).

It is unexpected that retatrutide failed to increase FOC in LA. However, we also noted before that glucagon failed to increase FOC in LA (Neumann et al. 2023a; Schmidt et al. 2024). Likewise, others failed to detect a PIE of glucagon in the left atrium of the adult rat (Merino et al. 2015). Hence, one possibility is that in the LA, the mRNA in the cardiomyocytes for the GCGR is not translated into a protein. Others reported in the rat right atrium that the mRNA of the GCGR is much more highly expressed in the sinus node (that is part of the upper RA) than in the remainder of the right atrium (Merino et al. 2015). We speculate that the same may hold true for the expression of the GCGR in the mouse right atrium, and this might, in part, explain our findings. One can furthermore understand why retatrutide is unable to exert a FOC in the left atrium base by a similar reasoning. It is reported that the expression of the GCGR is much less in the LA of mice than in the RA of mice (Ali et al. 2014). Hence, the density of the GCGR in LA might simply be too low to be stimulated by retatrutide. One could test our hypothesis by using mice with deletion of the GCGR only in sinus node cells, only in the remainder of the RA, only in the LA, or only in the ventricle but this is beyond the scope of the present initial study on retatrutide.

The protein expression of the GCGR in the mouse cardiac regions cannot be readily tested at present because GCGR specific antibodies are not well characterized. Many signals in the literature may result from unspecific Western blots. A similar argument can be made for the lack of any effect via GLP-1 or GIPR which we have put forward elsewhere (Neumann et al. 2024a). Another possibility that merits consideration is that the GIPR and the GLP-1 receptor may be present on non-cardiomyocytes such as fibroblasts or smooth muscle cells or endothelial cells in the atrium of the mouse.

One might alternatively speculate that GLP-1 and GIP are expressed and functional in RA and LA but that they increased cAMP in different compartments of the mouse right and left atrial cardiomyocytes that do not couple to FOC or beating rate. Alternatively, CGGR might fail to couple to adenylyl cyclase in LA and in the RA outside the sinus node. In other words, functional GCGR (that can be stimulated by retatrutide) might be present in the sinus node of the mouse heart but not in other parts of the RA. We also assume that PCE of retatrutide are not GIPR and neither GLP-1R-mediated because the PCE of retatrutide was not blocked by 100 nM exendin9-39 nor by 100 nM ProGIP, drugs that at these concentrations blocked the PIE of semaglutide or GIP in the human atrium (Neumann et al. 2024a, b a; Neumann et al. 2024b). The selective effect of retatrutide on beating rate but not force of contraction is not without precedence: prostaglandin E1 increased beating rate in the kitten heart but failed to increase FOC in the kitten heart (Kaumann and Birnbaumer 1974).

One word might be useful on the possible physiological role of adenosine receptors and M-cholinoceptors for the pharmacological role of retatrutide. Adenosine and acetylcholine are formed in the mammalian heart (Burnstock 2017, Lewartowski and Mackiewicz 2015). Under stressors like coronary ischemia and reperfusion adenosine and acetylcholine are released from cardiac cells. Hence, these receptors might be beneficial to suppress detrimental positive chronotropic effects in patients. But this could be the subject of clinical studies by others.

The fact that we failed to block the PCE of retatrutide by propranolol argues that retatrutide does not act via release of cardiac noradrenaline; in other words, that retatrutide act as an indirect sympathomimetic drug. The indirect sympathomimetic effect occurs also in the mouse heart, as we have shown e.g. the anorectic agent amphetamine or for the hallucinogenic drug MDMA (Neumann et al. 2023c, d). There are early reports that glucagon might increase FOC in the canine heart via release of endogenous noradrenaline or that glucagon might directly stimulate β-adrenoceptors (Farah and Tuttle 1960). Our data exclude such an interpretation at least in the mouse heart: we suggest instead that retatrutide increased the beating rate in LA not via release of noradrenaline and subsequent stimulation of adrenoceptors but instead because retatrutide stimulated GCGR in the sinus node of the mouse (Fig. 10). The situation might be different in vivo. There are clinical studies wherein injection of glucagon raised plasma concentrations of noradrenaline in patients (Kuchel et al. 1985). Hence, one cannot rule out that retatrutide might increase noradrenaline levels in the plasma of mice (and patients) when retatrutide were injected. However, under our experimental conditions, this seems not be an issue.

As concerns further the mechanism of action, we tried to obtain some insights from phosphorylation experiments. If retatrutide acts in the left or right atrium to increase cAMP, then retatrutide should increase the phosphorylation state of phospholamban (Fig. 1). However, this was not the case (100 nM retatrutide, data not shown), while isoprenaline increased the phosphorylation state of phospholamban in RA and LA as we reported often before (Gergs et al. 2010, 2019). Hence, cAMP levels and PKA activity lead to phospholamban phosphorylation and may cause the positive inotropic effects of β-adrenergic stimulation (e.g., Gergs et al. 2010). In contrast, retatrutide only increases beating rate in RA but neither in LA not in RA increases FOC (a decline in force is reported here). Hence, we might argue that fittingly, phospholamban phosphorylation is not elevated after retatrutide in LA and RA. Does that mean that phospholamban phosphorylation is not involved in the PCE of retatrutide? Not necessarily. It may simply be that phospholamban phosphorylation is only increased in the sinus node cells, because GCGR are only present and/or only active in sinus node cells and the number of sinus node cells in the whole RA is too small to lead to a detectable increase of phospholamban phosphorylation. However, the exact mechanism in the mouse sinus node of retatrutide remains to be elucidated, e.g., using isolated sinus node cells from mice.

There are data from clinical studies that GLP-1 receptor agonists, like retatrutide, may exert antiarrhythmic effects in the atria (Bode et al. 2024). It is not evident whether these beneficial effects of GLP-1 agonists are secondary to weight loss or due to direct cardiac actions. If one assumes that obesity leads to a higher incidence of arrhythmias, then a reduction of weight should lower the incidence of arrhythmias (Bode et al. 2024). One might also speculate that GLP1-agonists are directly antiarrhythmic drugs. Current thinking is that GLP-1 agonists increase cAMP in the heart (e.g., Wallner et al. 2015). Moreover, many papers claim that all cAMP increasing agents increase the incidence of arrhythmias (Tamargo et al. 2012). Admittedly these data refer to the huge heart failure studies and might not be valid without any exception (Tamargo et al. 2012). Hence, one has to wait for good clinical trials in non-obese patients with atrial fibrillation and find out whether in them a reduction of arrhythmias occurs with GLP1-receptor agonists.

In summary, we can now address the hypotheses raised in the Introduction in this way: retatrutide failed to raise the FOC in LA or in RA. Retatrutide augmented the beating rate via GCGR and not via GLP-1R or GIPR.

## Data Availability

All source data for this work (or generated in this study) are available upon reasonable request.
